# Heterogeneous Associations Between Community Social Capital and Loneliness: A Cross-sectional Study in 2019

**DOI:** 10.2188/jea.JE20250020

**Published:** 2026-01-05

**Authors:** Qiuyi Liu, Koryu Sato, Naoki Kondo

**Affiliations:** 1 Department of Social Epidemiology, Graduate School of Medicine and School of Public Health, Kyoto University, Kyoto, Japan; 2 Faculty of Policy Management, Keio University, Kanagawa, Japan

**Keywords:** loneliness, older adults, community social capital, sociodemographic subgroups

## Abstract

**Background:**

Loneliness is prevalent among older adults and is linked to physical and mental health problems. Community social capital has been suggested to mitigate its impact, but its heterogeneity across socioeconomic groups has not been explored.

**Methods:**

We analysed cross-sectional data from the 2019 Japan Gerontological Evaluation Study (JAGES) with 24,206 participants aged 65 years or older. Loneliness was measured using University of California, Los Angeles’s 3-item scale, and community social capital was assessed using civic participation, social cohesion, and reciprocity. Modified Poisson regression models were used, adjusting for sociodemographic factors and individual responses to the questions on social capital. Interaction effects of gender, education, and income were examined.

**Results:**

Higher levels of community social cohesion (prevalence ratio [PR] 0.84; 95% confidence interval [CI], 0.75–0.94) and community reciprocity (PR 0.64; 95% CI, 0.51–0.80) were inversely associated with loneliness. The relationship between community civic participation and loneliness varied by educational attainment. Interaction analysis indicated that individuals with higher education levels (≥13 years) who engaged in community civic participation had a lower prevalence of loneliness (PR 0.74; 95% CI, 0.57–0.95) compared to those with lower education levels. No clear interactions were observed for gender or income.

**Conclusion:**

Community social capital, particularly social cohesion and reciprocity, was associated with lower levels of loneliness among older adults. The effect of civic participation differed by education, showing a stronger negative association among individuals with higher education levels (≥13 years). Tailored interventions accounting for educational backgrounds are needed while promoting social capital universally.

## INTRODUCTION

Loneliness is prevalent among older adults, who are particularly vulnerable to this condition.^1^ Age-related changes and losses, declining health, changes in family structure, and fewer social connections contribute to an increased risk of loneliness among this demographic.^2^ According to the World Health Organization (WHO), health is defined as “a state of complete physical, mental, and social well-being, and not merely the absence of disease or infirmity.”^3^ Loneliness, though not explicitly part of this definition, is closely linked to social well-being and is increasingly recognized as an important public health concern. It can be understood as a barrier to achieving the full state of health as defined by the WHO. Moreover, studies have shown that loneliness is associated with several physical and mental illnesses, including diabetes, coronary heart disease, cancer, and depression, underscoring its significance as a critical dimension of health.^4^ It has also been identified as a predictor of functional decline and mortality.^5^^,^^6^ A study on the impact of loneliness on total life expectancy and health expectancy in older adults revealed that those who experienced loneliness had shorter lives and fewer healthy years than their non-lonely counterparts.^7^ Consequently, addressing and reducing loneliness among older adults is crucial for enhancing their overall quality of life and health.

Various protective factors for loneliness, such as having a partner, robust social networks, engaging in social activities, and managing depression, are being studied to enhance the health and well-being of older adults.^8^ In recent years, the continued advancement of research on social capital has led to its increasing use in public health research. Social capital can be broadly defined as “resources that are accessed by individuals as a result of their membership of a network or a group.”^9^ These resources include both structural aspects—such as social networks and participation in community activities—and cognitive dimensions, including trust, mutual reciprocity, and shared norms. Existing literature highlights that components of social capital, such as trust, social connections, and opportunities for social participation, play a critical role in reducing loneliness among older adults.^10^^,^^11^ Increased social participation and social support are linked with subsequent reductions in loneliness and improvements in quality of life among older adults.^12^

Previous studies, including those conducted in China, Finland, and Spain, have examined the association between social capital and loneliness in later life, often emphasizing both structural and cognitive aspects of social engagement.^10^^–^^12^ While these studies provide important insights, most have assessed social capital at the individual level, and relatively few have incorporated community-level perspectives or examined how these associations may vary by socioeconomic factors.

Nonetheless, individual-level and community-level social capital differ in their stability and scope.^13^ Individual-level social capital, defined as resources embedded in personal social networks, varies based on individual circumstances, such as the size and activity of one’s social ties.^14^ In contrast, community-level social capital emphasizes collective attributes of a community, such as trust, reciprocity, and social cohesion, which are relatively stable and widespread.^15^ This distinction highlights the importance of addressing both levels of social capital to alleviate loneliness effectively. Although the potential of community-level social capital in reducing loneliness is promising, limited evidence exists on whether its effects differ across individual socioeconomic characteristics. Further research is needed to clarify these heterogeneous associations and inform more equitable interventions.

Moreover, social capital fosters enhanced social cohesion and mutual support (reciprocity) within communities, which can benefit all individuals, including more vulnerable groups such as those with lower education and income levels.^9^ The positive effects of social connections and support extend beyond directly engaged participants, thereby supporting the well-being of the entire community, including its most vulnerable members. Even people who lack direct group connections can experience a protective effect on mental health.^16^ This benefit underscores the importance of studying community-level social capital, which clarifies its contextual effect as a group attribute or collective property that individual-level analyses may fail to capture.

Existing research indicates that the relationship between community social capital and health may vary across different communities and socioeconomic contexts. Therefore, further analysis of these differences is needed to understand the underlying mechanisms of its impact better.^17^ While there is potential for positive relationships between community social capital and loneliness for some subpopulations, there could also be negative associations for others. For instance, Haseda et al identified that civic participation, a component of community social capital (ie, community contexts promoting social participation), was associated with an increased risk of depressive symptoms in older adults with low income.^18^ Similarly, Amemiya et al showed that community social capital was associated with the improvement of functional ability, but this largely depended on individual psychosocial characteristics and gender.^19^ Given that the impact of community social capital can be inconsistent across different socioeconomic groups, it is critical to understand its heterogeneity, particularly as it relates to disadvantaged groups, to promote equitable public health interventions in the community.

Therefore, the purpose of this study was to investigate the heterogeneous association between community social capital, as measured by its three components (ie, civic participation, social cohesion, and reciprocity), and loneliness in Japanese older adults across different sociodemographic subgroups.

## METHODS

### Data

We used cross-sectional data from the 2019 wave of the Japan Gerontological Evaluation Study (JAGES). The JAGES was designed to assess social determinants of health among older Japanese adults. In the JAGES 2019 wave, anonymous self-administered questionnaires were mailed to functionally and cognitively independent participants aged 65 years or older.

We mailed self-reported questionnaires to older adults in 61 municipalities from November 2019 to January 2020. We invited 45,934 individuals, and a total of 31,857 people responded, corresponding to a response rate of 69.4%. We excluded invalid responses (*n* = 6,175) and missing answers to loneliness (*n* = 1,476), and 24,206 were included in the final analysis.

This study was reviewed and approved by the ethics committees at Kyoto University (R3153-2), Chiba University (2493), and the National Center for Geriatrics and Gerontology (992).

### Measurements

#### Loneliness

We utilized the Japanese version of University of California, Los Angeles (UCLA)’s 3-item loneliness scale to measure loneliness.^20^ The scale includes three questions: “How often do you feel that you lack companionship?” “How often do you feel left out?” “How often do you feel isolated from others?” with answers ranging from “hardly ever (1), some of the time (2), and often (3).” Participants with a total score of 3–5 were considered not lonely, whereas those with a score of 6–9 were considered lonely. This classification is based on previous studies using the UCLA 3-item Loneliness Scale, where scores of 6–9 have been grouped as “lonely”.^21^

#### Community social capital

Community social capital was evaluated using the Health-Related Social Capital Scale developed and validated among older Japanese people.^15^ The scale comprised three dimensions: civic participation, social cohesion, and reciprocity. Based on the conceptual framework proposed by Berkman et al, civic participation was categorized as a structural component of social capital, while social cohesion and reciprocity were regarded as cognitive components.^9^

Civic participation refers to participation in five types of community-based group activities: volunteering, sports, hobbies, cultural activities, and teaching groups. Social cohesion was assessed using the following questions: “Do you think that people living in your area can be trusted, in general? Do you think that people living in your area try to help others in most situations? How attached are you to your area?” The sum of the percentage of respondents who answered “very” and “moderately” to the questions counted in the score, other answers were “neutral,” “slightly,” and “not at all,” these answers were not counted in the score. Reciprocity was measured using the following questions: “Is there someone who listens to your concerns and complaints? Do you listen to others’ concerns and complaints? Do you have someone who looks after you when you are sick and confined to a bed for a few days?” The sum of the percentage of respondents who received support from any persons counted in the score.

The average scores for civic participation, social cohesion, and reciprocity were calculated for each elementary school district, as the validated scale did.^15^ The elementary school district is a reasonable unit to measure community social capital because it often represents an administrative unit of a former village and because many local activities (eg, senior citizens’ clubs, agricultural cooperatives, and local festivals) take place within each of them.

#### Potential sources of heterogeneity and covariates

In our regression model, age, gender, equivalized household income (low [>3 million Japanese yen {JPY}], middle [2–3 million JPY], high [>3 million JPY]), educational attainment (≤9 years, 10–12 years, ≥13 years), marital status (married, single/divorced/widowed/other), employment (unemployed, employed), and individual responses to the questions on social capital (individual-level civic participation, social cohesion, and reciprocity) were adjusted as confounding factors. Individual-level social capital was derived from the same questionnaire items used to construct community-level measures but were based on each respondent’s own answers. Civic participation, social cohesion, and reciprocity were each coded as binary variables, indicating the presence or absence of the corresponding aspect of social capital at the individual level.

To explore heterogeneity in the association between community social capital and loneliness, we consider gender, education, and income as potential sources of heterogeneity.

### Statistical analysis

A modified Poisson regression model was used to investigate the association between community social capital and loneliness.^22^ In model 1, age and gender were included. In model 2, age, gender, income, educational attainment, marital status, employment and individual-level social capital were added.

To investigate the potential heterogeneity in the associations between community social capital and loneliness, we assessed the modifying effects of gender, educational attainment, and income on these relationships. Heterogeneous associations were assessed by introducing interaction terms into the main regression model to estimate multiplicative interactions, as well as calculating the additive interaction through the relative excess risk due to interaction (RERI).

Missing data on covariates and effect modifiers were handled using multiple imputation by chained equations under the assumption of missing at random. 10 imputed datasets were generated using appropriate regression models depending on the variable type. The outcome variable was not imputed, and participants with missing outcome data were excluded from the analysis. Estimates were combined using Rubin’s rules.

To address the potential bias from excluding participants with missing loneliness data, we conducted a sensitivity analysis by performing multiple imputation of the outcome variable (loneliness) using the same imputation model as before. The imputation model included all covariates used in the main analysis, as well as additional variables related to loneliness. The main analysis model was then re-fitted using these imputed datasets to examine the robustness of the main results. Both prevalence ratios (PRs) and RERI were calculated for each imputed dataset. Rubin’s rules were applied to combine the estimates across the imputed datasets.

All analyses were performed using STATA (version 17.0; StatCorp, College Station, TX, USA).

## RESULTS

Table 1 presents the respondents’ descriptive characteristics. We analyzed 24,206 participants with an average age of 74.7 years, and 51.8% were females. The score of community civic participation was 0.95 (the possible range of values was between 0 and 5), and those of community social cohesion and community reciprocity were 2.05 and 2.84, respectively (the possible range of values was between 0 and 3). Of the participants, 3,745 (15.5%) reported loneliness.

**Table 1.** Descriptive characteristics of participants (*n* = 24,206)

	*n* (%) or mean [SD]
**Gender**	
Male	11,657 (48.2)
Female	12,549 (51.8)
**Age, years**	
65–69	5,773 (23.8)
70–74	7,032 (29.1)
75–79	5,934 (24.5)
80–84	3,582 (14.8)
≥85	1,885 (7.8)
**Income**	
Low (<2 million JPY)	10,459 (43.2)
Middle (2–3 million JPY)	8,266 (34.2)
High (>3 million JPY)	2,553 (11.06)
Missing	2,928 (12.1)
**Education**	
≤9 years	6,019 (24.9)
10–12 years	10,296 (42.5)
≥13 years	7,348 (30.3)
Missing	543 (2.24)
**Marital status**	
Married	17,473 (72.2)
Single/divorced/widowed/other	6,431 (26.6)
Missing	302 (1.25)
**Employment**	
Unemployed	15,822 (65.4)
Employed	6,798 (28.0)
Missing	1,586 (6.6)
**Individual-level civic participation**	
No participation	11,326 (46.8)
Any participation	10,698 (44.2)
Missing	2,182 (9.0)
**Individual-level social cohesion**	
Not cohesive	3,209 (13.3)
Cohesive	20,767 (85.8)
Missing	230 (0.9)
**Individual-level reciprocity**	
No support	1,176 (4.9)
Any support	22,680 (93.7)
Missing	350 (1.4)
**Community-level social capital**	
Civic participation	0.95 [0.30]
Social cohesion	2.05 [0.27]
Reciprocity	2.84 [0.12]
**Loneliness**	
Feel loneliness	3,745 (15.5)
Not feel	20,461 (84.5)

JPY, Japanese yen; SD, standard deviation.

The results of the modified Poisson regression analysis are presented in Table 2. In the crude model and model 1, all the three dimension of social capital was associated with a lower prevalence of loneliness. Even after fully adjusting for covariates in model 2, higher levels of community social cohesion (PR 0.84; 95% confidence interval [CI], 0.75–0.94) and community reciprocity (PR 0.64; 95% CI, 0.51–0.80) were associated with a lower prevalence of loneliness, whereas the association of community civic participation was attenuated.

**Table 2.** Prevalence ratio for loneliness determined using modified Poisson regression analysis (*n* = 24,206)

Variables	Null model	Model 1	Model 2
	PR (95% CI)	PR (95% CI)	PR (95% CI)
**Community civic participation**	0.75 (0.68–0.82)	0.76 (0.68–0.84)	0.95 (0.86–1.05)
**Community social cohesion**	0.68 (0.61–0.76)	0.67 (0.61–0.75)	0.84 (0.75–0.94)
**Community reciprocity**	0.53 (0.42–0.67)	0.54 (0.43–0.67)	0.64 (0.51–0.80)

CI, confidence interval; PR, prevalence ratio.

Model 1 adjusted age and gender; model 2 is fully adjusted.

**Figure 1.**
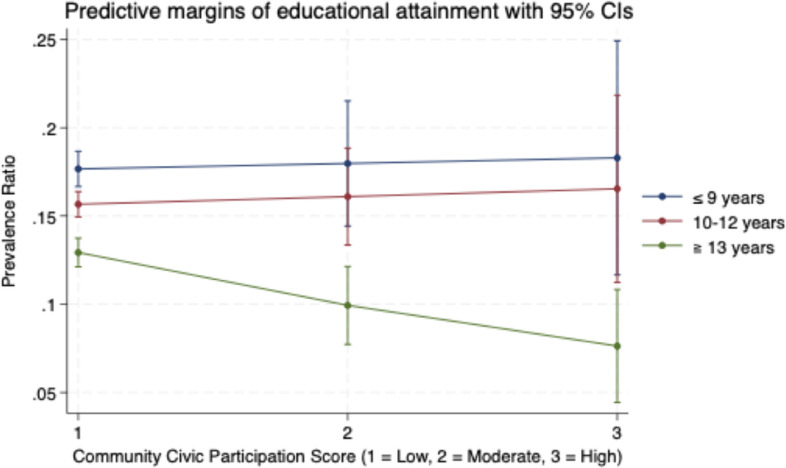
Predictive margins of educational attainment on community civic participation

Table 3 presents the results of the additive and multiplicative interaction analysis between the three dimensions of community social capital (civic participation, social cohesion, and reciprocity) and educational attainment, income, and gender. For the additive interaction effects, there was no evidence of interactions across the subgroups. However, a notable multiplicative interaction was observed for individuals with higher education levels (≥13 years) interacting with community civic participation, which was associated with lower levels of loneliness (PR 0.74; 95% CI, 0.57–0.95). This trend is further illustrated in Figure 1 (see below), which shows the predicted prevalence of loneliness across community civic participation levels stratified by educational attainment. Community civic participation was modeled as a continuous variable. For interpretability, predictive margins were calculated at three fixed values: 1 (low), 2 (moderate), and 3 (high). As seen in the figure, loneliness levels decrease more significantly for individuals with higher education as community civic participation increases. No other multiplicative interactions were detected.

**Table 3.** Additive and multiplicative interaction effects of community social capital dimensions by education, income, and gender

	Additive interaction	Multiplicative interaction
	
	RERI (95% CI)	PR (95% CI)
Community CP × Education (ref: 10–12 years)		
≤9 years	0.04 (−0.60 to 0.68)	0.99 (0.78–1.24)
≥13 years	−0.22 (−0.54 to 0.10)	0.74 (0.57–0.95)
Community SC × Education (ref: 10–12 years)		
≤9 years	−0.23 (−1.09 to 0.64)	1.12 (0.87–1.42)
≥13 years	0.16 (−0.32 to 0.65)	1.13 (0.86–1.49)
Community R × Education (ref: 10–12 years)		
≤9 years	0.40 (−0.58 to 1.38)	0.82 (0.49–1.36)
≥13 years	−0.28 (−2.19 to 1.64)	0.87 (0.50–1.51)
Community CP × Income (ref: Middle [2–3 million JPY])		
Low (<2 million JPY)	0.20 (−0.31 to 0.70)	0.96 (0.77–1.21)
High (>3 million JPY)	−0.03 (−0.60 to 0.54)	0.97 (0.64–1.47)
Community SC × Income (ref: Middle [2–3 million JPY])		
Low (<2 million JPY)	0.30 (−0.26 to 0.86)	0.94 (0.74–1.20)
High (>3 million JPY)	−0.48 (−1.91 to 0.96)	0.76 (0.46–1.25)
Community R × Income (ref: Middle [2–3 million JPY])		
Low (<2 million JPY)	0.70 (−0.20 to 1.61)	0.74 (0.44–1.23)
High (>3 million JPY)	0.32 (−1.48 to 2.12)	1.61 (0.51–5.12)
Community CP × Gender (ref: male)		
Female	−0.50 (−1.16 to 0.17)	1.08 (0.89–1.31)
Community SC × Gender (ref: male)		
Female	−0.87 (−1.93 to 0.19)	0.93 (0.76–1.13)
Community R × Gender (ref: male)		
Female	−1.78 (−4.00 to 0.44)	0.89 (0.58–1.37)

CI, confidence interval; CP, civic participation; R, reciprocity; RERI, relative excess risk due to interaction; PR, prevalence ratio; SC, social cohesion.

All models are adjusted for age, gender, income, educational attainment, marital status, employment, participation in a social group, individual social cohesion, and individual social support.

As a sensitivity analysis, we performed multiple imputation that included the loneliness outcome variable, using the same imputation model as in the main analysis. The descriptive characteristics of the imputed dataset remained generally consistent with the complete-case dataset (eTable 1). The prevalence of loneliness in the imputed dataset was estimated to be similar to that in the complete-case dataset.

The PR estimates from the imputed dataset were also consistent with the complete-case analysis. However, when examining additive interactions (ie, RERI) using the imputed dataset, we observed substantial instability in some subgroups, particularly among individuals with lower educational attainment (see eTable 2 and eTable 3).

## DISCUSSION

Using data from a nationwide survey of Japanese older adults, this study showed that community social capital, particularly community social cohesion and community reciprocity were negatively associated with the prevalence of loneliness, emphasising the potential value and the importance of these factors. The study found that these associations between community social capital and loneliness were largely consistent across different socioeconomic groups, with the exception of civic participation, which was more strongly associated with lower levels of loneliness among individuals with higher education levels (≥13 years) compared to those with lower education levels.

Our results suggested that community social cohesion and reciprocity are inversely associated with loneliness. The concept of social capital can be divided into structural and cognitive dimensions, and social cohesion and reciprocity are classified as cognitive dimensions.^23^ These findings are consistent with previous research indicating the importance of cognitive social capital for the prevention of loneliness in older adults.^11^ However, our study extends the relationship between community-level social capital in loneliness among older adults. High social cohesion within a community fosters a sense of belonging and mutual support. Additionally, the long-term attachment of older adults to their community environment can also alleviate feelings of loneliness.^5^ This is particularly relevant for older adults, who may experience reduced social networks and mobility. According to the measurements, reciprocity involves emotional support, such as having someone to listen to their worries and concerns. This emotional support is crucial for older adults, knowing that they can receive support from community members when needed provides a sense of security and belonging.

These findings are consistent with previous research, which observed largely homogeneous associations between community-level social capital and well-being outcomes across gender and socioeconomic subgroups.^24^ Although we did not observe significant interaction effects by gender, previous studies have identified gendered differences in both the engagement with and the health effects of community social capital. For instance, women are generally more likely to participate in social activities than men in Japan, which may lead to differences in exposure or benefits.^24^ Moreover, studies have suggested that the influence of social capital components such as cohesion or participation on health outcomes may vary by gender.^19^^,^^25^ These discrepancies might reflect differences in social roles, expectations, or psychosocial mechanisms. It is also possible that our findings differ due to the subjective nature of the outcome (loneliness), or due to the limitations of the cross-sectional design, which may not fully capture temporal or cumulative effects. Further longitudinal research is needed to better understand how gender moderates the association between social capital and health outcomes in older adults.

Building upon these observations, prior studies have demonstrated that higher levels of social capital in communities are not only associated with better health outcomes but also hold the potential to mitigate socioeconomic health disparities.^26^ The consistency of these associations across different demographic groups suggests that investments in community social capital could be an efficient public health strategy to address loneliness, as they have the potential to benefit a broad range of individuals without solely relying on targeted interventions based on socioeconomic status.

Our findings of the multiplicative interaction of civic participation and educational attainment on loneliness, higher education might provide individuals with better communication skills, larger social networks, and greater confidence in engaging in community activities. Research indicates that older individuals with lower levels of education may face barriers to participating in various social activities, which limits their opportunities for community engagement and the associated benefits.^1^ However, previous studies have shown that participation, particularly in leadership or managing roles, can sometimes lead to negative consequences for older adults with lower education levels. For example, Ashida et al reported that taking on such roles may impose additional stress and burdens, potentially impacting health outcomes.^27^ Moreover, education has been shown to improve the use of technologies, which can further facilitate social engagement by providing older adults with the tools to connect with others and access social resources.^28^

In addition to educational disparities, older adults from low-income backgrounds may also face structural barriers to participating in community-based activities. Although many community-based programs, such as volunteer or hobby groups, aim to enhance social participation, prior research suggests that they may disproportionately attract individuals with higher financial and educational status. For example, Kawaguchi et al found that structured social activity programs in serviced housing were primarily utilized by financially advantaged older adults.^29^ Similarly, Tsuji et al reported significantly lower participation rates in volunteer activities among low-income and low-education older adults in Japan, underscoring the need for inclusive program design that ensures access for disadvantaged populations.^30^

As technology continues to evolve, it becomes an important medium for social participation, making it easier, more efficient, and more enjoyable for older adults to stay connected. Therefore, promoting lifelong learning and providing access to educational programs can serve as a vital strategy to enhance social participation and improve the overall well-being of older adults, particularly among those who may otherwise be excluded due to socioeconomic disadvantage.

To address potential bias arising from excluding participants with missing loneliness data, we conducted a sensitivity analysis where the loneliness outcome was also included in the multiple imputation model. This imputation approach follows recommendations for reducing selection bias when outcome data are missing.^31^ However, there were substantial instabilities observed in some RERI estimates from the imputed dataset, particularly in subgroups with lower education levels. Given this instability, we decided to present the RERI estimates from the complete-case analysis as the main interaction results, while the PR estimates from the imputed dataset including the loneliness are presented as a sensitivity analysis in the supplementary section. This approach allows us to maintain consistency in the primary findings while also acknowledging the potential impact of imputing the outcome variable.

There are several limitations to this study. First, the method used to measure loneliness may not capture the full complexity of the concept, as it relies on subjective self-reporting, which can be influenced by individual differences in interpreting loneliness. Different measurement methods can complicate comparisons between studies. For instance, distinct measures of loneliness capture different characteristics of people who suffer from it, showing only a 45% overlap between individuals classified as lonely by direct questioning and by the UCLA loneliness scale.^32^ This highlights the need for more comprehensive and standardized loneliness measurements to improve the accuracy of findings.^33^ Second, the generalizability of the findings may be constrained due to the focus on a Japanese population that was not nationally representative. This demographic and cultural specificity limits the applicability of the results to broader populations. Third, the exclusion of certain variables could lead to confounding bias, hindering an inclusive understanding of the studied relationships. The study also overlooked detailed analyses of how marital transitions or regional differences might influence community social capital and loneliness. Furthermore, it did not identify how different types of civic participation affect older adults across educational levels, highlighting an area for more in-depth future investigations.

### Conclusion

This study highlights the association between community social capital, particularly social cohesion and reciprocity, and lower levels of loneliness among older adults. The findings indicate that the association of civic participation with loneliness is stronger among individuals with higher educational attainment (≥13 years) compared to those with lower education levels. These insights can guide the design of targeted interventions to address loneliness, especially for disadvantaged subgroups. This includes implementing universal interventions that are adjusted in scale and intensity according to the level of social vulnerability. For example, prioritizing support for individuals with lower education levels can help bridge gaps in civic participation. Future research should validate these findings and identify effective strategies to promote social capital in communities, ensuring that interventions are customized to meet diverse needs.

## SUPPLEMENTARY MATERIAL

eTable 1. Descriptive characteristics of participants (n = 25,682)

eTable 2. Prevalence ratios for loneliness determined using modified Poisson regression analysis (n = 25,682)

eTable 3. Additive and multiplicative interaction effects of community social capital dimensions by education, income, and gender
