# Supplementary material for: Heterogeneous Associations Between Community Social Capital and Loneliness: A Cross-sectional Study in 2019
Source: J Epidemiol. 2026 Jan 5;36(1):28–34. doi: 10.2188/jea.JE20250020 (PMC12698323; doi:10.2188/jea.JE20250020)
Supplement: Supplementary file 1 [file je-36-028-s001.pdf]

eTable 1. Descriptive characteristics of participants (n=25,682)

	Respondents with loneliness data	Respondents with missing loneliness data
	n (%) or mean [SD]	n (%) or mean [SD]
**Gender**		
Male	11,657 (48.2)	582 (39.4)
Female	12,549 (51.8)	894 (60.6)
**Age, years**		
65–69	5,773 (23.8)	224 (15.2)
70–74	7,032 (29.1)	314 (21.3)
75–79	5,934 (24.5)	366 (24.8)
80–84	3,582 (14.8)	290 (19.7)
≥85	1,885 (7.8)	282 (19.1)
**Income**		
Low (<2 million JPY)	10.459 (43.2)	599 (62.1)
Middle (2–3 million JPY)	8,266 (34.2)	281 (29.1)
High (>3 million JPY)	2,553 (11.06)	84 (8.7)
Missing	2,928 (12.1)	512 (34.7)
**Education**		
≤9 years	6,019 (24.9)	536 (36.3)
10–12 years	10,296 (42.5)	452 (30.6)
≥13 years	7,348 (30.3)	287 (19.4)
Missing	543 (2.24)	201 (13.6)
**Marital status**		
Married	17,473 (72.2)	872 (59.1)
Single/divorced/widowed/other	6,431 (26.6)	436 (29.5)
Missing	302 (1.25)	168 (11.4)
**Employment**		
Unemployed	15,822 (65.4)	379 (25.7)
Employed	6,798 (28.0)	93 (6.3)
Missing	1,586 (6.6)	1,004 (68.0)
**Individual-level civic participation**		
No participation	11,326 (46.8)	609 (41.3)
Any participation	10,698 (44.2)	474 (32.1)
Missing	2,182 (9.0)	393 (26.6)
**Individual-level social cohesion**		
Not cohesive	3,209 (13.3)	251 (17.0)
Cohesive	20,767 (85.8)	1,114 (75.5)
Missing	230 (0.9)	111 (7.5)
**Individual-level reciprocity**		
No support	1,176 (4.9)	83 (5.62)
Any support	22,680 (93.7)	1,213 (82.2)
Missing	350 (1.4)	180 (12.2)
**Community-level social capital**		
Civic participation	0.95 [0.30]	0.78 [0.33]
Social cohesion	2.05 [0.27]	2.03 [0.27]
Reciprocity	2.84 [0.12]	2.83 [0.11]
**Total**		
Feel loneliness	24,206	1,476

JPY, Japanese yen; SD, standard deviation.

eTable 2. Prevalence ratios for loneliness determined using modified Poisson regression analysis （n=25,682）

Variables	Crude model	Model 1	Model 2
	PR (95% CI)	PR (95% CI)	PR (95% CI)
**Community civic participation**	0.75 (0.68–0.83)	0.75 (0.63–0.77)	0.95 (0.86–1.06)
**Community social cohesion**	0.70 (0.63–0.78)	0.70 (0.61–0.75)	0.88 (0.75–0.98)
**Community reciprocity**	0.52 (0.42–0.66)	0.53 (0.42–0.67)	0.64 (0.51–0.80)

CI, confidence interval; PR, prevalence ratio.

Crude model: unadjusted model including only the exposure variables; Model 1 adjusted age and gender, Model 2: additionally adjusted for income, educational attainment, marital status, employment, participation in a social group, individual social cohesion, and individual social support.

eTable 3. Additive and multiplicative interaction effects of community social capital dimensions by education, income, and gender

	Additive interaction	Multiplicative interaction
	RERI (95% CI)	PR (95% CI)
Community CP×Education (ref: 10–12 years)		
≤9 years	-3.64 (-67.9 to 60.6)	1.09 (0.34–3.39)
≥13 years	-0.14 (-0.35 to 0.07)	0.80 (0.64–0.99)
Community SC×Education (ref: 10–12 years)		
≤9 years	-193.7 (-2.39e+03 to 2004.3)	2.45 (0.84–7.21)
≥13 years	0.11 (-0.30 to 0.52)	1.04 (0.82–1.34)
Community R×Education (ref: 10–12 years)		
≤9 years	0.80 (-1.23 to 2.83)	0.54 (0.05–6.02)
≥13 years	-0.06 (-1.32 to 1.19)	0.94 (0.56–1.55)
Community CP×Income (ref: Middle[2–3 million JPY])		
Low (<2 million JPY)	0.20 (-0.31 to 0.70)	0.94 (0.75–1.17)
High (>3 million JPY)	0.12 (-1.32 to 1.56)	1.03 (0.68–1.58)
Community SC×Income (ref: Middle[2–3 million JPY])		
Low (<2 million JPY)	0.30 (-0.26 to 0.86)	0.88 (0.69–1.12)
High (>3 million JPY)	-0.53 (-2.02 to 0.96)	0.78 (0.42–1.28)
Community R×Income (ref: Middle[2–3 million JPY])		
Low (<2 million JPY)	0.70 (-0.20 to 1.61)	0.73 (0.44–1.23)
High (>3 million JPY)	-0.04 (-2.87 to 2.79)	1.56 (0.51–4.83)
Community CP×Gender (ref: male)		
Female	0.32 (-0.44 to 1.08)	1.11 (0.92–1.34)
Community SC×Gender (ref: male)		
Female	-0.15 (-0.69 to 0.39)	0.93 (0.75–1.14)
Community R× Gender (ref: male)		
Female	-0.64 (-2.80 to 1.51)	0.82 (0.52–1.28)

CI, confidence interval; CP, civic participation; PR, prevalence ratio; R, reciprocity; RERI, relative excess risk due to interaction; SC, social cohesion.

All models are adjusted for age, gender, income, educational attainment, marital status, employment, participation in a social group, individual social cohesion, and individual social support.
